# Strategies for managing EMA/CO resistant in gestational trophoblastic neoplasia a systematic review and meta analysis

**DOI:** 10.1007/s12672-025-03707-5

**Published:** 2025-10-14

**Authors:** Febia Erfiandi, Setyo Teguh Waluyo, Candra Novi Ricardo Sibarani, Gatot Nyarumenteng Adhipurnawan Winarno, Aini Sofa Haniah, Nicholas Adrianto

**Affiliations:** 1 Division of Gynecologic Oncology, Department of Obstetrics and Gynecology, Padjadjaran University, Dr. Hasan Sadikin General Hospital, Bandung, Indonesia; 2 Division of Gynecologic Oncology, Department of Obstetrics and Gynecology, Lambung Mangkurat University, Ulin General Hospital, Banjarmasin, Indonesia; 3 School of Medicine and Health Sciences, Atma Jaya Catholic University of Indonesia, Pluit Raya No. 2, Penjaringan, North Jakarta, Daerah Khusus Ibukota, Jakarta, 14440 Indonesia

**Keywords:** Chemotherapy, Gestational trophoblastic neoplasia, Meta-analysis, Systematic review

## Abstract

**Objective:**

This systematic review and meta-analysis evaluate the efficacy and safety of salvage regimens in managing EMA/CO-resistant GTN, providing evidence to inform optimal treatment strategies.

**Methods:**

A literature search was conducted in PubMed, ScienceDirect, Cochrane Library, Google Scholar, and Wiley Online Library until December 27, 2024. Studies on EMA/CO chemoresistance in gestational trophoblastic neoplasia (GTN) were included, and alternative regimens and surgical interventions were also considered. Exclusion applied to non-human studies and those unrelated to EMA/CO chemoresistance. Data extraction and quality assessment followed PRISMA, Cochrane ROB-2, and the Newcastle-Ottawa Scale. A meta-analysis was performed using a random-effects model, with heterogeneity (I²) and publication bias assessed. The study was registered with PROSPERO (CRD42024574582).

**Results:**

Eight studies met the inclusion criteria, encompassing patients predominantly with advanced-stage (FIGO III-IV) and high-risk GTN. EMA/EP and EP/EMA were the most frequently evaluated salvage regimens, with a pooled complete remission rate of 78.7% (95% CI: 67.4–88.1%) across 84 patients. No significant heterogeneity (I² = 27.05%) or publication bias was detected. Alternative regimens, including BEP, FAEV, and TP/TE, demonstrated favourable remission rates in small cohorts but lacked generalizability. Neutropenia (68%), thrombocytopenia (41%), and anaemia (30%) were the most commonly reported toxicities with EP/EMA. Safety data for other regimens were limited.

**Conclusion:**

EMA/EP and EP/EMA remain the most effective and well-studied salvage regimens for EMA/CO-resistant GTN, demonstrating high remission rates with manageable toxicity. While alternative regimens such as BEP, FAEV, and TP/TE show encouraging results, their limited evidence base precludes definitive comparison. Further prospective studies are needed to establish optimal salvage strategies and refine toxicity management.

## Introduction

Gestational trophoblastic neoplasia (GTN) is a rare malignancy arising from placental trophoblastic cells, encompassing invasive mole, choriocarcinoma, placental site trophoblastic tumor (PSTT), and epithelioid trophoblastic tumor (ETT) [1, 2]. Despite their rarity, GTN is highly curable with appropriate chemotherapy, emphasizing the importance of early diagnosis and individualized treatment [3]. GTN is typically treated with the EMA-CO regimen, which includes etoposide, methotrexate, actinomycin D, cyclophosphamide, and vincristine [4]. However, 30% to 40% of patients may develop resistance or experience relapse after initial remission, necessitating salvage chemotherapy [5]. High-risk GTN, defined as having a FIGO prognostic score of 7 or higher, poses challenges due to its aggressive nature, potential for metastasis, and chemoresistance [6]. Resistance to EMA/CO therapy is marked by plateauing or rising β-hCG levels, new metastases, or disease progression [5]. For patients with high-risk GTN who do not respond to initial multi-agent chemotherapy, salvage therapy using platinum/etoposide-based regimens, often combined with surgical interventions and brain radiation, has proven to be highly effective. This treatment strategy significantly improves survival and outcomes in patients with high-risk GTN [7].

Salvage regimens, such as EMA/EP (etoposide, methotrexate, actinomycin D, and cisplatin), have shown promising efficacy, with success influenced by disease stage, extent of metastases, and baseline β-hCG levels [8]. Predictive factors for treatment failure, including high FIGO scores and extensive metastases, remain critical in guiding therapeutic decisions. Targeted therapies and novel protocols are being explored to overcome chemoresistance, although managing persistent high-risk GTN remains challenging. This systematic review and meta-analysis aim to evaluate the efficacy of salvage strategies for EMA/CO-resistant GTN, focusing on remission rates, survival outcomes, and associated toxicities to guide optimal management. This systematic review and meta-analysis evaluate the efficacy of EMA/CO-resistant GTN management strategies, analyzing remission rates, survival outcomes, and toxicities to guide optimal care.

## Methods

### Literature search

A comprehensive literature search was conducted using the Pubmed, Science Direct, Cochrane Library, Google Scholar, and Wiley Online Library databases. Searches were conducted from database inception to December 27th, 2024. The search strategy included Medical Subject Headings (MeSH) terms and keywords such as “Gestational Trophoblastic Neoplasia,” “EMACO,” “Chemoresistance,” and “Management.” Boolean operators were used to combine these terms (e.g., “AND,” “OR”) to refine results. No restrictions were applied to language or publication year during the search process.

### Selection criteria

This review included studies involving patients with gestational trophoblastic neoplasia (GTN) who developed chemoresistance to EMA/CO, defined as persistent elevation of serum β-hCG levels despite treatment, in contrast to recurrent disease characterized by re-elevation after prior normalization. The intervention of interest was salvage chemotherapy, including but not limited to EMA/EP and TP/TE regimens. Although no direct comparator was required, eligible studies were those reporting remission outcomes—specifically, the achievement of complete remission, defined as normalization of serum β-hCG to < 5 mIU/mL—following second-line treatment. Studies involving non-human subjects or lacking data on EMA/CO-resistant cases were excluded.

### Data extraction

Four authors (FE, CNRS, ASH, NA) independently conducted the search, screened articles using specified keywords, and removed duplicates. Articles were then shortlisted based on title/abstract. Full-texts meeting eligibility were thoroughly reviewed for data synthesis, with exclusion reasons noted. Discrepancies were settled by a fourth party (GNAW). Three authors independently evaluated all abstracts, with conflicts resolved by CNRS. If multiple studies shared participants and outcomes, excluding others, the most detailed study was chosen to avoid redundancy. STW assessed the articles’ quality independently. Our research followed PRISMA, Cochrane ROB-2, and Newcastle-Ottawa Scale [9–11].

### Statistical analyses

Meta-analysis of proportions was conducted using logit transformations and a random-effects model, with pooled proportions calculated via MedCalc (v19.5.1) [12]. A P-value of < 0.05 was considered statistically significant. Heterogeneity was measured using I²; studies with I² <50% used a fixed-effects model, while higher heterogeneity warranted a random-effects model. Publication bias was assessed using Egger’s test and Begg’s rank correlation. The study protocol was registered with the PROSPERO international registry (CRD42024574582).

## Results

**Fig. 1:**
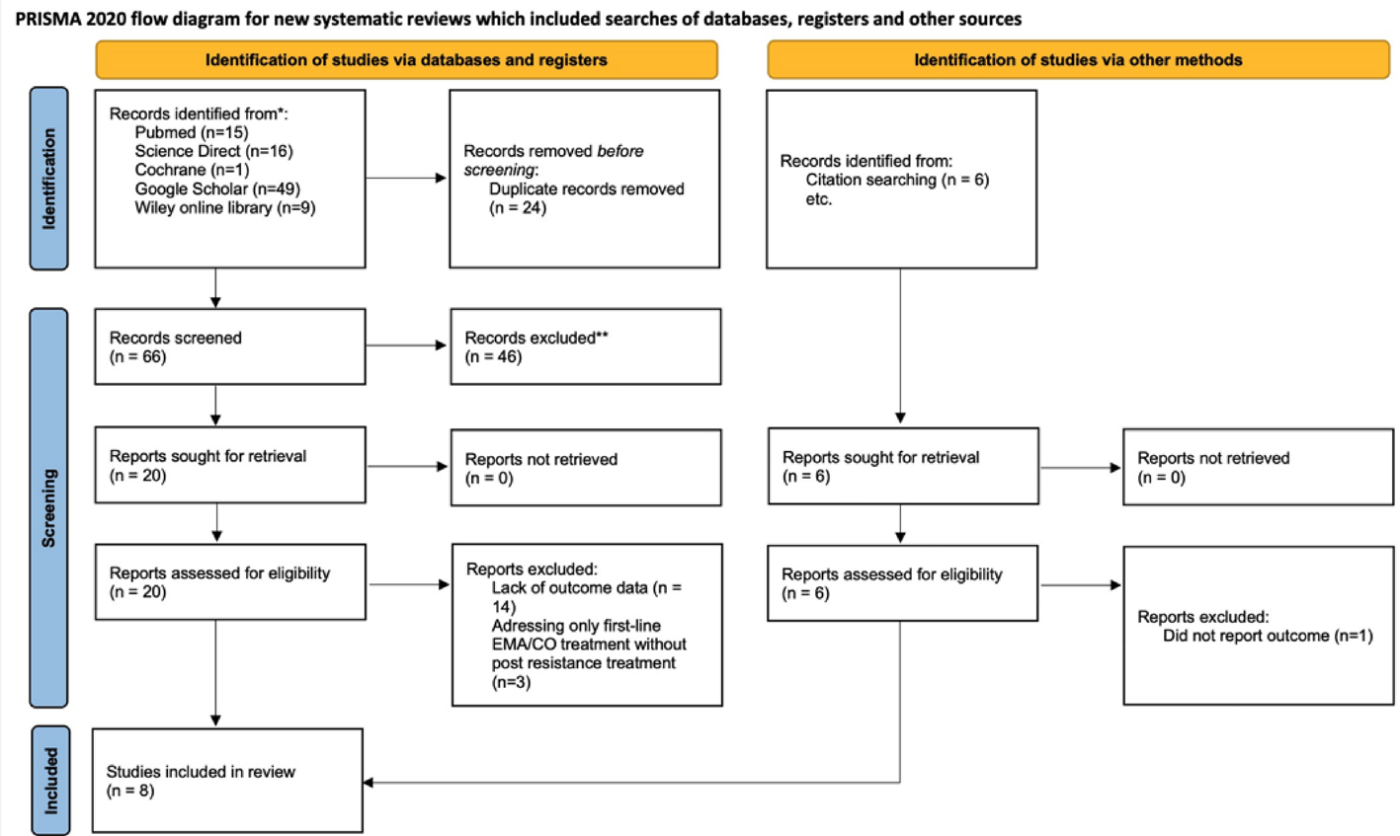
PRISMA flow diagram [10]

The initial literature search identified 66 potential articles. After screening titles and abstracts, eight articles underwent detailed review, with eight ultimately meeting the inclusion criteria for meta-analysis (Fig. 1). The median patient age across these studies ranged from 25 to 39 years. The majority presented with advanced-stage GTN (FIGO stages III-IV), and high-risk prognostic scores (≥ 8) were common in approximately 65% of cases. The predominant salvage chemotherapy regimens evaluated were EP-EMA and EMA-EP, though some studies reported alternative regimens like FAEV, BEP, and TP/TE (Table 2). Adjuvant surgery was utilized in approximately 25% to 50% of cases, primarily addressing localized disease or treatment-related complications. 

### Risk of bias

**Fig. 2:**
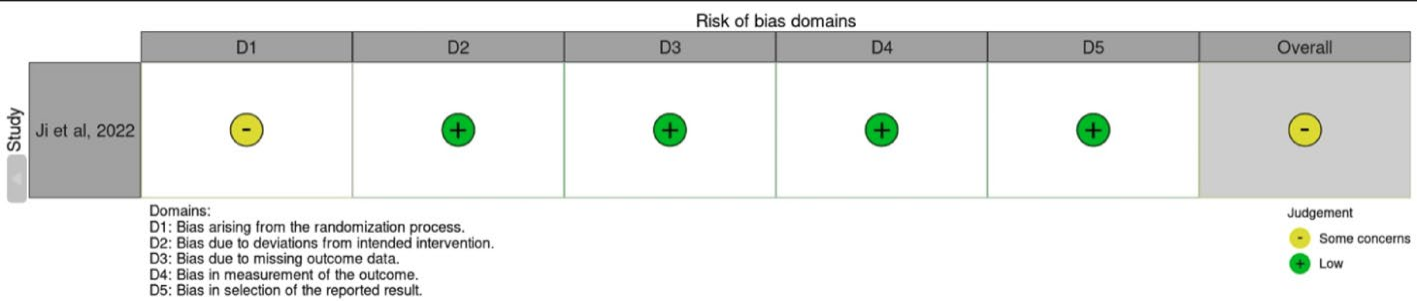
Risk of bias assessment cochrane ROB-2 [9]

The NOS assessment for the studies shows overall low to moderate risk of bias in most studies (Table 1). The ROB-2 assessment for Ji et al. indicates a low risk of bias across most domains, except for some concerns regarding the randomization process, leading to an overall judgment of moderate reliability (Fig. 2) [13].

**Table 1:** Risk of bias - Newcastle Ottawa scale [11]

No	Author	Selection	Comparability	Outcome	Risk of bias
1	Newlands et al., 2000 [14]	★★★★	★★	★★★	Low
2	Mao et al., 2007 [18]	★★★★	★★	★★★	Low
3	Lurain et al., 2004 [1]	★★★★	★	★★	Low
4	Pastoriza-Alcaraz et al., 2021 [8]	★★★★	★	★★	Low
6	Lu et al., 2008 [22]	★★★★	★★	★★★	Low
7	Wang J et al., 2008 [16]	★★★★	★★	★★★	Low
8	Lurain et al., 2012 [7]	★★★	★	★★	Moderate

### Complete remission

**Fig. 3:**
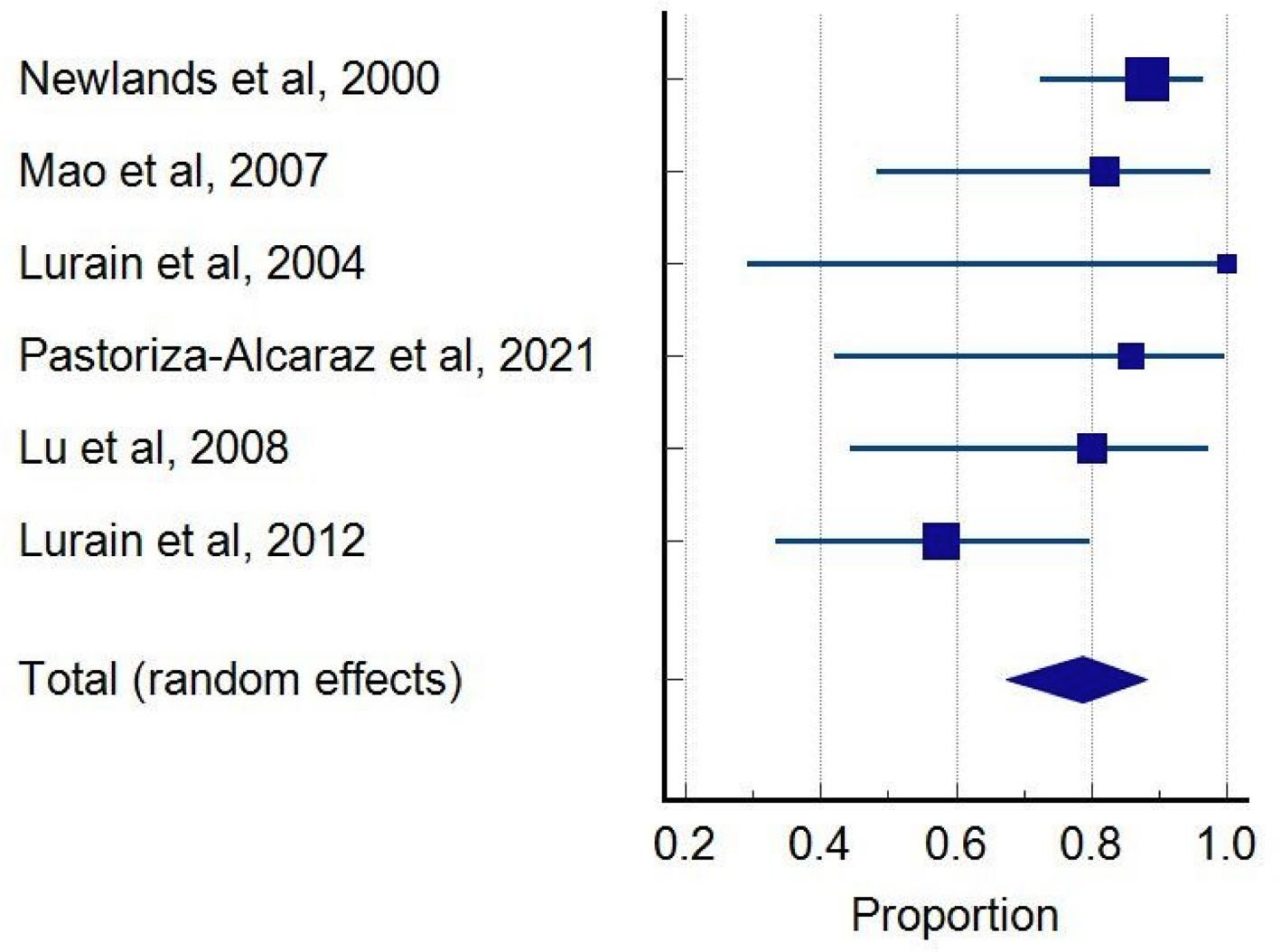
A pooled proportion of nine studies reporting complete remission patients with EMA/CO resistance received EMA-EP regimen as salvage chemotherapy [8, 14, 15, 18, 22–25]

Our review predominantly identified the EP-EMA / EMA-EP regimen as the most frequently evaluated salvage chemotherapy for EMA/CO-resistant GTN, complete remission rate was calculated at 0.787 (95% CI: 0.674–0.881), indicating a 78.7% remission rate across the EMA-EP group (*N* = 84) (Fig. 3). Statistical analysis revealed no evidence of publication bias, as demonstrated by Begg’s and Egger’s intercept tests. No significant heterogeneity was observed between studies (Q = 6.8541, *P* = 0.2317, I²=27.05%). Alternative regimens such as BEP, FAEV, and TP/TE demonstrated promising preliminary outcomes but were supported by single studies with limited patient numbers, significantly constraining their generalizability and comparability (Table 3).

### Safety

Neutropenia was the most commonly reported adverse event, affecting approximately 68% of patients receiving EP-EMA [8, 14]. Thrombocytopenia and anemia were also notable, occurring in 41% and 30% of patients, respectively. Gastrointestinal disturbances, including nausea and vomiting, were observed in 40% of cases, while mild hepatotoxicity was occasionally reported [8]. Comprehensive toxicity data for alternative regimens such as BEP, FAEV, and TP/TE were either limited or not reported, precluding robust comparative safety analysis (Table 4).

**Table 2:** Characteristics of included studies

No	Author	Country	Study design	Number of patients	Age (years)	FIGO stage	Prognostic score	Salvage regimen
1	Newlands et al., 2000 [14]	United Kingdom	Retrospective study	34	18–47	III-IV	High risk (22)	EP-EMA
2	Di Mao et al., 2007 [18]	China	Retrospective study	11	25–58	III-IV	12 (5–20)	EP-EMA
3	Lurain et al., 2004 [1]	China	Retrospective study	10	19–43	III-IV	12 (5–22)	EMA-EP/BEP
4	Pastoriza-Alcaraz et al., 2021 [8]	Philippines	Retrospective study	20	30	III-IV	7–12 : 9 > 12 : 11	EP-EMA
5	Ji M et al., 2022 [13]	China	Randomized controlled trial	8	35.5	III-IV	-	FAEV
6	Lu et al., 2008 [22]	China	Retrospective study	12	26.8	III-IV	-	EMA-EP
7	Wang J et al., 2008 [16]	United Kingdom	Retrospective study	8	27 (18–48)	III-IV	-	TP/TE
8	Lurain et al., 2012 [7]	USA	Retrospective study	19	30 (21–56)	III-IV	12 (8–20)	EMA-EP

FIGO = The International Federation of Gynecology and Obstetrics; EP-EMA = etoposide and cisplatin with etoposide, methotrexate, and dactinomycin; FA = 5-fluorouracil and actinomycin D regimen; BEP = bleomycin; FAEV = oxuridine, actinomycin D, etoposide and vincristine

**Table 3:** Efficacy of included studies

No	Author	Salvage Regimen	Number of Patients	CR (%)	OS (%)	Adjuvant surgery
1	Newlands et al., 2000 [14]	EP-EMA	34	30/34 (80)	-	Yes (22 ops in 17 pts: hysterectomy, thoracotomy, craniotomy)
2	Mao et al., 2007 [18]	EP-EMA	11	9/11 (81.8)	-	Yes (8 patients; 7/8 achieved CR)
3	Lurain et al., 2004 [15]	EP-EMA	3	3/3 (100)	2/3 (67)	Yes (not limited to EMA/CO resistant)
		BEP	7	6/7 (87)	-	
4	Pastoriza-Alcaraz et al., 2021 [8]	EP-EMA	7	6/7 (86)	4/6 (57)	Not reported
5	Ji M et al., 2022 [13]	FAEV	8	8/8 (100)	-	Not reported
6	Lu et al., 2008 [22]	EP-EMA	10	8/10 (80)	-	Yes (Not limited to EMA/CO resistant)
7	Wang J et al., 2008 [16]	TP/TE	7	6/8 (75)	-	Not reported
8	Lurain et al., 2012 [7]	EMA-EP	19	11 (58)	9 (47)	Yes (No seperable cohort data)

EP-EMA = etoposide and cisplatin with etoposide, methotrexate, and dactinomycin; FA = 5-fluorouracil and actinomycin D regimen; BEP = bleomycin; FAEV = oxuridine, actinomycin D, etoposide and vincristine; CR = Complete remission; OS = Overall survival

**Table 4:** Safety of included studies

No	Study	Regimen	*n*	Neutropenia (*n*)	Thrombocytopenia (*n*)	Anemia (*n*)	GI symptoms (%)	Other toxicities (*n*)
1	Newlands et al., 2000 [14]	EP-EMA	22	15	9	NA	NA	NA
2	Mao et al., 2007 [18]	EP-EMA	11	NA	NA	NA	NA	NA
3	Lurain et al., 2004 [1]	EMA-EP/BEP	10	NA	NA	NA	NA	NA
4	Pastoriza-Alcaraz et al., 2021 [8]	EP-EMA	20	2 (Grade III) 9 (Grade IV)	3 (Grade III) 2 (Grade IV)	6 (Grade II-IV)	8 (40)	Hypokalemia (2) Grade III-IV
5	Ji M et al., 2022 [13]	FAEV	8	NA	NA	NA	NA	NA
6	Lu et al., 2008 [22]	EMA-EP	NA	NA	NA	NA	NA	NA
7	Wang J et al., 2008 [16]	TP/TE	8	NA	NA	NA	NA	NA
8	Lurain et al., 2012 [7]	EP-EMA	19	NA	NA	NA	NA	NA

NA = Not available; GI = Gastrointestinal; EP-EMA = etoposide and cisplatin with etoposide, methotrexate, and dactinomycin; FA = 5-fluorouracil and actinomycin D; BEP = bleomycin; FAEV = Floxuridine, actinomycin D, etoposide and vincristine

## Discussion

Our systematic review and meta-analysis evaluated several salvage chemotherapy regimens for managing EMA/CO-resistant GTN. EMA-EP/EP-EMA, which consists of etoposide, methotrexate, actinomycin D, and cisplatin, emerged as the most commonly studied regimen, demonstrating a pooled CR rate of 78.7%. This favorable efficacy positions EMA-EP as a reliable therapeutic option, reflecting its status as the standard salvage chemotherapy regimen after EMA/CO failure. Alternative salvage regimens evaluated in single studies included BEP, FAEV, and TP/TE. BEP exhibited an 85.7% CR rate (6/7 patients), FAEV showed a 100% CR rate (8/8 patients), and TP/TE demonstrated a 75% CR rate (6/8 patients). Despite promising results, these regimens require cautious interpretation due to their limited patient numbers and absence of additional confirmatory studies. Further investigation through larger, prospective trials is necessary before these alternative regimens can be confidently recommended.

Salvage chemotherapy for EMA/CO-resistant GTN involves several regimens with varying degrees of efficacy. EMA-EP remains the most widely used option, with CR rates ranging from 60% to 90%, depending on the extent of disease resistance and tumor burden. BEP (bleomycin, etoposide, and cisplatin) achieves a CR rate of approximately 100% but demonstrates limited effectiveness in ultra-high-risk cases [15]. The FAEV regimen (floxuridine, actinomycin D, etoposide, and vincristine) has shown comparable efficacy to EMA/CO, with an 100% CR rate as salvage regimen [13]. Similarly, TP/TE (paclitaxel/cisplatin alternating with paclitaxel/etoposide) demonstrates a CR rate of 75% in heavily pretreated or relapsed patients [16]. These findings highlight EMA-EP as the standard salvage option, with FAEV and TP/TE serving as effective alternatives in specific patient subsets [13, 16]. The prognostic significance of factors such as pre-salvage β-hCG levels, FIGO stage, and prognostic scoring was evident, with higher remission rates observed in patients with limited metastatic disease and lower β-hCG levels at baseline [8, 17]. Adjuvant surgery emerged as a critical component of management in select cases, particularly in patients with localized resistant disease or complications and for patients ԝho desire future childbearing. Some studies reported improved outcomes with the integration of surgical interventions, emphasizing the importance of a multidisciplinary approach [4, 8, 18, 19].

The safety profiles of salvage regimens are critical in guiding treatment choices for EMA/CO-resistant GTN. Salvage chemotherapy inherently carries a significant toxicity burden, posing challenges to treatment adherence and overall outcomes. These regimens require a delicate balance between managing adverse effects and maintaining efficacy. EMA-EP, while effective, is associated with moderate hematologic toxicity, including grade 3–4 neutropenia in 45% of patients and thrombocytopenia in 20%, which aremanageable with supportive care [8, 14, 18, 20, 21]. BEP, despite its efficacy, has significant toxicity, with severe renal and hematologic effects, including neutropenia (68%) and thrombocytopenia (40%), making it less favorable for ultra-high-risk patients [15]. The FAEV regimen demonstrated higher rates of myelosuppression (60.9%) but with predictable and manageable toxicity when supported by granulocyte-colony stimulating factor (G-CSF) [13]. TP/TE, in contrast, exhibits a more favorable safety profile, with 42% of patients experiencing transient grade 3–4 neutropenia and no reported cases of neutropenic sepsis [16]. It is also associated with reduced treatment delays and fewer dose modifications compared to EP/EMA. Overall, EMA-EP remains the preferred choice due to its balance of efficacy and manageable toxicity, while TP/TE offers a less toxic alternative for heavily pretreated or intolerant patients. Further studies are needed to refine these regimens and tailor them to individual patient needs. Gastrointestinal toxicities, including nausea, vomiting, and diarrhea, were also widely reported, with an incidence as high as 85% [18]. These were generally manageable with antiemetics and supportive care, but their high prevalence highlights the need for patient education and anticipatory symptom management. Hypokalemia and hypomagnesemia were significant concerns in patients receiving EP-EMA, with up to 40% affected in studies like necessitating regular electrolyte monitoring and supplementation [8, 22].

Anemia and thrombocytopenia, while less frequent than neutropenia, still posed challenges in a subset of patients. Severe cases requiring blood transfusion or dose modifications were reported [8, 20, 21]. Hepatotoxicity, though generally mild, was observed in a minority of patients, particularly in emphasizing the need for liver function monitoring during treatment [18, 20]. These findings emphasize the need for individualized toxicity management plans, particularly for high-risk patients with significant comorbidities or extensive disease burden [23].

The limitations of the analyzed studies are predominantly linked to their retrospective nature, small sample sizes, and variability in defining chemoresistance and treatment outcomes. The heterogeneity observed in EMA/CO-resistant GTN treatment outcomes can be attributed to several factors. Baseline disease burden plays a critical role, with patients exhibiting extensive metastases or high β-hCG levels at the initiation of salvage therapy often demonstrating poorer outcomes. Selection of salvage therapy may also shaped by center-level protocols and drug availability (e.g., access to actinomycin D, platinum agents, paclitaxel), supportive-care capacity (e.g., G-CSF, transfusion/electrolyte monitoring), and radiotherapy resources; these contextual differences likely influenced regimen choice, dose intensity, treatment delays, and observed outcomes, but were inconsistently reported and could not be adjusted for. Particularly in low-resource countries where access to key drugs and supportive care is limited. Treatment protocols contribute to variability, as differences in chemotherapy regimens, dosing schedules, and supportive care measures significantly influence efficacy and toxicity profiles. Additionally, the inconsistent application of adjunctive therapies, such as surgery and radiotherapy, introduces further complexity, highlighting the need for standardized approaches to optimize patient outcomes. Furthermore, the lack of RCT comparing salvage regimens limits the generalizability of the findings, underscoring the need for robust prospective studies​.

## Conclusion

Treatment of EMA/CO-refractory GTN continues to pose a clinical challenge, requiring salvage chemotherapy strategies to balance efficacy with tolerable toxicity. EMA-EP currently serves as the salvage regimen of choice, with acceptable remission rates and fairly comparable outcomes in multiple studies. Other options, including BEP, FAEV, and TP/TE, show promising initial efficacy but require further studies through more extensive, strictly designed prospective trials. To optimize patient care, management should be individualized by FIGO risk, metastatic burden/sites, fertility goals, with proactive toxicity stewardship to maintain treatment intensity, multidisciplinary strategies, unifying salvage chemotherapy, targeted surgical intervention, and active, supportive care are necessary. Surgery and radiotherapy can aid control in focally refractory or high-risk disease but are best considered as co-interventions. Future studies must continue to accord importance to comparative effectiveness trials, customized toxicity evaluation strategies, fertility preservation, and patient quality-of-life enhancements toward affording better therapeutic modalities to this rare yet highly aggressive malignancy.

## Data Availability

All data generated or analysed during this study are included in this published article.
